# Pacing Behaviour Development and Acquisition: A Systematic Review

**DOI:** 10.1186/s40798-022-00540-w

**Published:** 2022-12-09

**Authors:** Stein Gerrit Paul Menting, Andrew Mark Edwards, Florentina Johanna Hettinga, Marije Titia Elferink-Gemser

**Affiliations:** 1 Department of Human Movement Sciences, University Medical Center Groningen, University of Groningen, Groningen, PO Box 196, 9700 AD The Netherlands; 2 School of Psychology and Life Sciences, Canterbury Christ Church University, Canterbury, UK; 3 Department of Sport, Exercise and Rehabilitation, Faculty of Health and Life Sciences, Northumbria University, Room 238, Northumberland Building, Newcastle Upon Tyne, NE1 8ST UK

**Keywords:** Pacing, Skill, Development, Junior, Acquisition, Experience, Sports, Exercise

## Abstract

**Background:**

The goal-directed decision-making process of effort distribution (i.e. pacing) allows individuals to efficiently use energy resources as well as to manage the impact of fatigue on performance during exercise. Given the shared characteristics between pacing behaviour and other skilled behaviour, it was hypothesized that pacing behaviour would adhere to the same processes associated with skill acquisition and development.

**Methods:**

PubMed, Web of Science and PsycINFO databases between January 1995 and January 2022 were searched for articles relating to the pacing behaviour of individuals (1) younger than 18 years of age, or (2) repeatedly performing the same exercise task, or (3) with different levels of experience.

**Results:**

The search resulted in 64 articles reporting on the effect of age (*n* = 33), repeated task exposure (*n* = 29) or differing levels of experience (*n* = 13) on pacing behaviour. Empirical evidence identifies the development of pacing behaviour starts during childhood (~ 10 years old) and continues throughout adolescence. This development is characterized by an increasingly better fit to the task demands, encompassing the task characteristics (e.g. duration) and environment factors (e.g. opponents). Gaining task experience leads to an increased capability to attain a predetermined pace and results in pacing behaviour that better fits task demands.

**Conclusions:**

Similar to skilled behaviour, physical maturation and cognitive development likely drive the development of pacing behaviour. Pacing behaviour follows established processes of skill acquisition, as repeated task execution improves the match between stimuli (e.g. task demands and afferent signals) and actions (i.e. continuing, increasing or decreasing the exerted effort) with the resulting exercise task performance. Furthermore, with increased task experience attentional capacity is freed for secondary tasks (e.g. incorporating opponents) and the goal selection is changed from achieving task completion to optimizing task performance. As the development and acquisition of pacing resemble that of other skills, established concepts in the literature (e.g. intervention-induced variability and augmented feedback) could enrich pacing research and be the basis for practical applications in physical education, healthcare, and sports.

## Key Points

- Pacing behaviour develops during childhood and adolescence, as individuals gain the capability to appreciate that the distribution of effort leads to increased exercise task performance.
- Gaining experience allows an exerciser to refine the match between their performance capabilities and the task demands, resulting in pacing behaviour that better fits the task demands (i.e. task characteristics and environmental factors) facilitating increased exercise task performance.
- Future research should investigate the exciting idea of applying lessons from the skill acquisition and development literature to aid individuals’ pacing behaviour and as a result enhance their exercise performance.

## Background

Humans are unable to sustain high-intensity physical work indefinitely and thus exercise performance has limitations, of which the causes are diverse depending on the specific activity [1]. Sustained physical work over a defined performance duration has been shown to result in either an involuntary decline in motor skill execution or requires an increasing effort to maintain performance level [2]. These phenomena are interlinked with changes in sensations that regulate the physiological integrity of the exerciser, such as localized pain, nausea and heat stress, which collectively represent the concept of fatigue [2]. To deal with these phenomena in a sports setting, the exerciser needs to manage the balance of exertion required to successfully complete the task’s goal, with an optimal distribution of energy resources adapted to the duration of the event [1, 3]. This balances the power losses needed to overcome velocity-related frictional forces, and power production [4] while avoiding premature deterioration of motor skill performance due to overwhelming or catastrophic fatigue [5]. Achieving this balance is of particular relevance in technical sports such as speed skating [6]. In order to perform this feat, exercisers engage in a process of decision-making regarding how and when to exert effort to successfully complete physical tasks [7, 8]. At a fundamental level, the continuous decision to be made by the exerciser is whether to increase, decrease or continue exerting the same level the effort [9]. This decision is influenced by factors such as the exercise task characteristics (e.g. exercise duration [10] and biomechanical traits [6]) and the environment (e.g. presence of opponents [11] or temperature [12]), in combination with afferent signals from the musculoskeletal system [1]. This goal-directed decision-making process regulating the distribution of effort over a predetermined exercise task has been defined as pacing [7, 13]. Given that humans are not entirely rational decision-makers [14], factors like motivation [15], mood [16], and self-efficacy [17] impact the decision-making and the subsequent task performance. It is important to state that the self-regulatory elements of the pacing process are thought to be cyclical; the experience that is gained with each iteration of the task is used to recalibrate the informed decision-making for the next task execution [18].

Pacing fits the description of a skill, as it is task-specific, goal-directed behaviour that is improved with increased training and experience [19, 20]. Skills are often investigated at a behavioural level; the study of skilled behaviour is concerned with quantification of the extent to which a given behaviour achieved the goal that was intended or instructed [19]. When considering pacing in this context, the outcome of the goal-directed decision-making process regarding the distribution of effort could therefore be defined as ‘pacing behaviour’. Quantifying pacing behaviour has generally been achieved by plotting an outcome measure for effort (e.g. power output) over time [21, 22].

The view of pacing as a skill reflects that it goes through development and has to be acquired [23, 24]. Skill development encompasses the effect of age, specifically in maturing children and adolescents, on skilled behaviour [19]. Any real-world exercise task necessarily entails both cognitive and motor components, which undergo drastic development during childhood and adolescence [25]. To illustrate, on average, the adolescent growth spurt starts at approximately 9 years of age in girls and about 11 years of age in boys, with a peak height velocity at an average age of 12 and 14 years old, for girls and boys, respectively [26, 27]. Physical attributes which play a key role during exercise, such as total lung capacity, alveolar surface, stroke volume and cardiac output of the heart, and muscle mass develop accordingly [28, 29]. Additionally, the period between 10 and 24 years old is distinguished by a reorganization of the neural circuitry of the higher brain centres [30, 31]. The higher brain centre to develop most during this period is the prefrontal cortex, the area of the brain associated with abstract thinking, planning and decision-making [31, 32]. Neurological evidence suggests that the prefrontal cortex is essential to pacing as it is said to facilitate the integration of afferent feedback into top-down control of motor unit recruitment [33]. As pacing encompasses a complex psychophysiological process [1, 34], it seems more than likely that it develops throughout childhood and adolescence [1, 18, 35]. Developing the skill to adequately pace an exercise task is crucial in an individual's development as it provides a feeling of competence, motivating children and adolescents to engage more in exercise, with all the associated health benefits in later life [1]. Vice versa, inadequate development of pacing behaviour could negatively impact exercise performance while also affecting individuals’ long term exercise practices, health and well-being [35, 36]. A repeated inaccuracy in the distribution of effort during repeated exercise tasks over a longer period of time could lead to task overexertion, which could result in overtraining, injuries, burn-out and disincentivization to exercise, eventually causing drop-out from exercise and physical activity [1, 36]. At an acute level, a sub-optimal development of pacing behaviour could also yield problems for populations who experience difficulty self-regulating effort [37], such as people with an intellectual impairment [38]. A better understanding of the pathway and underpinning mechanisms of pacing behaviour development would therefore be a valuable tool to aid children’s development [18, 35].

Skill acquisition is known to be the relatively permanent change in behaviour as a result of prior experience [19, 39]. It is thought that skill acquisition goes through phases [19, 25, 40]. Initially, learners focus mainly on associating stimuli and actions in order to achieve the task goal. As acquisition continues, the relationship between variations in behaviour and task performance is used as a recalibration of the skill: good strategies are maintained, and inappropriate ones are discarded. The late stage of skill acquisition is often evidenced by the level of automatization; the learner performs the task using less of their conscious attention, leaving cognitive capacity for the execution of secondary tasks. When categorizing pacing as a skill, it is logical to assume that similar processes underlie the process of learning how to pace an exercise task. This allows for the application of lessons from the skill acquisition literature in the field of pacing. Studying pacing in a skill acquisition framework could therefore not only provide valuable information to the ongoing discussion regarding the debated workings of the pacing process [15, 41] but also provide practical information to coaches and healthcare professionals who aim to correct or fine-tune an individual’s distribution of effort by means of practice, to improve physical activity performance in both sports or healthcare settings [21, 42].

The relation between pacing behaviour and various physiological [1, 13], biomechanical [43], psychological [44] and more recently neurological [45] variables has been extensively studied to gain a deeper understanding of the symbiotic relation between pacing behaviour and exercise task performance. However, the development of pacing behaviour during childhood and adolescence and the acquisition of the skill through experience have received limited attention, despite holding the promise of a wealth of theoretical knowledge and practical applications. This review, therefore, aims to investigate the development of pacing behaviour during childhood and adolescence as well as the acquisition of the skill through experience. To achieve this aim, the existing literature will be systematically analysed for the effect of age (up until 18 years old) and gathering experience on pacing behaviour. Recognizing the similarities between pacing behaviour and skilled behaviour, it is hypothesized that pacing behaviour would adhere to the same characteristics associated with skill acquisition and development. If this is indeed the case, lessons learned for skill acquisition and development could be used to enrich the field of pacing research with future research goals and form practical guidelines to improve exercise performance.

## Methods

The current systematic review will be restricted to pacing behaviour in a sports and exercise setting, including only articles investigating a healthy population (for more information on pacing behaviour in a healthcare setting we recommend the review of Abonie et al. [42]). Although the study of pacing behaviour has a valuable role in healthcare and rehabilitation settings such as when reacquiring skills after neurological injury [37, 42], the majority of literature investigating pacing behaviour is set in a sports science setting where competition and maximal effort trials are common. The sports laboratory environment is well suited as a basis for experimental research, as it facilitates a standardized approach to a physical performance task in a controlled setting, measured by validated and accurate equipment [46]. PubMed, Web of Science and PsycINFO databases were searched for literature pertaining to the development and acquisition of pacing behaviour. The following search strategy was used:

1. Sport [Mesh]
  AND
2. Pacing OR Pacing behaviour OR Pacing strategy OR Race analysis
  AND
3. Develop OR Learn OR Experience OR Novice OR Age OR Children OR Adolescence OR Junior OR Youth OR Boy OR Girl.

**Fig. 1:**
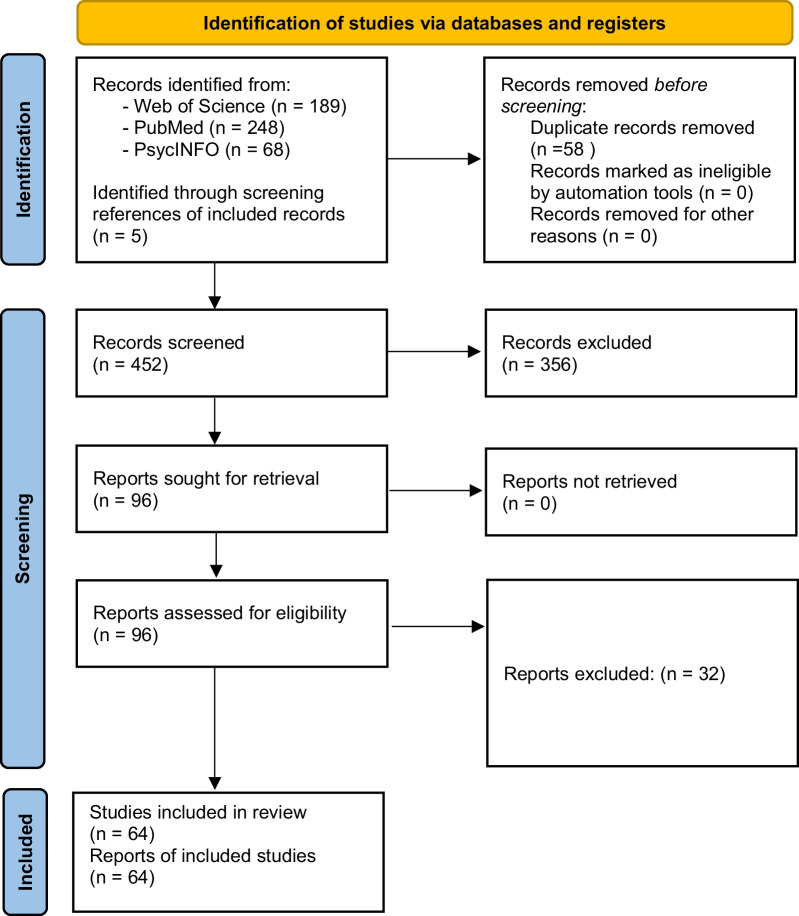
Flow diagram of the literature selection process with included articles (n) after each stage

Included articles had to be written in English, published between January 1995 and January 2022 and peer-reviewed. The option for human participants was selected for PubMed and PsycINFO; in Web of Science, (AND Human*) was added to line 1 of the search strategy. Following the literature on skill learning and development [19, 20], the included articles had to report on one or a combination of the following topics: the pacing behaviour of individuals younger than 18 years of age or the pacing behaviour of individuals repeatedly performing the same (or a very similar) exercise task or the effect of a period of practice on pacing behaviour (e.g. through a training program) or the comparison of pacing behaviour between groups with different levels of experience (i.e. novices vs. experts). To provide an extensive overview of the available literature, no selection was made regarding the type of exercise task (e.g. endurance, team-sport, resistance). Included articles had to quantify pacing behaviour by expressing a measure of effort (e.g. energy store depletion, power output, velocity) over a subset of the full exercise task (e.g. percentage of task completed). The initial search resulted in 505 articles (248 PubMed, 189 Web of Science, 68 PsycINFO). After eliminating duplicates, 447 articles remained. Screening the titles and abstracts, followed by screening the full text, led to exclusion of 388 articles, leaving 59 included articles (Fig. 1). To these included articles, the authors added five articles, which did not occur in the literature search, but instead were found through the reading of the introduction and discussion sections of included articles (specifically marked in Table 1). These five articles met the inclusion criteria and were deemed to yield valuable information regarding the aim of the current study.

**Table 1:** Overview of the included articles (n = 64), categorized by sport and distance

Study	Sport	Distance	No. of subjects (sex)	Age* (mean ± SD)	Repeated task exposure	Level of experience
Foster et al. [47]	Cycling	1500-m	9 (7 male)		Three trials, a minimum of 48 h apart	Experienced cyclists and speed-skaters (training 10 h/week)
Corbett et al. [48]	Cycling	2-km	9 (9 male)		Three trials within 2 week period	Novices
Menting et al. [49]	Cycling	2-km	10 (7 male)	15.8 ± 1.0	Four trials, within 6 weeks	Novices
Konings et al. [50]	Cycling	4-km	12 (?)		Four trials, 7 days apart	Experienced cyclists (≥ 2 years)
Ansley et al. [51]^a^	Cycling	4-km	7 (7 male)		Three consecutive trials, 17 min apart	Experienced cyclists (training 400–800 km/week)
Williams et al. [52]	Cycling	4-km	22 (22 male)		Two consecutive trials, 17 min apart	Novices
Mauger et al. [53]	Cycling	4-km	18 (18 male)		Four consecutive trials, 17 min apart	Experienced cyclists (training 11.5 ± 3.5 h/week, 1 competition per week)
Mauger et al. [54]	Cycling	4-km and 6-km	16 (16 male)		Four consecutive trials, 17 min apart	Experienced cyclists (training 12 ± 3 h/week, 1 competition per week)
Jones et al. [55]	Cycling	16.1-km	20 (20 male)		Three trials within 3 weeks (2–7 days apart)	Experienced cyclists (> 1 year)
Jeukendrup et al. [56]	Cycling	16-km	12 (12 male)		Two trials, 7–14 days apart	Experienced cyclists (training 3x/week, > 1 competition per year)
Boya et al. [57]	Cycling	16.1-km	20 (20 male)		Two trials within a 6–11-day period	∙ Experienced cyclists (14.1 ± 13 years, training 8.5 ± 2.1 h/week) ∙ Novices
Whitehead et al. [58]	Cycling	16.1-km	20 (20 male)			∙ Experienced cyclists (> 2 years) ∙ Novices
Martin et al. [59]^a^	Cycling	20-min (14.8 ± 0.6 km) (11.8 ± 0.6 km)	11 (11 male) 9 (9 male)			∙ More experienced cyclists (> 5 years) ∙ Less experienced cyclists (~ 2 years)
Marquet et al. [60]	Cycling	20-km	21 (21 male)		Two trials separated by a 1-week training program	Experienced cyclists (≥ 3 years, training ≥ 12 h/week)
Micklewright et al. [61]	Cycling	20-km	29 (29 male)		Three trials, 3–7 days apart	Experienced cyclists (> 2 years, 6.1 ± 5.2 years)
Hibbert et al. [62]	Cycling	20-km	30 (12 male)		Seven trials, minimum of 48 h apart	Novices
Schmit et al. [63]	Cycling	20-km	22 (22 male)		Two trials, 11 ± 4 days apart	Experienced triathletes (≥ 3 years, training 7 sessions/week)
Micklewright et al. [64]	Cycling Running	5-km 100-km	20 (15 male) 34 (32 male)			∙ Novice ∙ Experienced runners (5.6 ± 8.9 ultramarathons in past 2 years, training 61.4 ± 23.0 km/week)
Foster et al. [65]	A: Cycling B: Rowing C: Rowing D: Cycling	A: 3-km B: 2-km C: 2-km D: 10-km	?		A: six trials, 2–3 days apart B: three trials, 2–3 days apart C: Two sets of two trials, one month of training program apart D: three trials	Novices
Cerasola et al. [66]	Rowing	1000-m	96 (48 male)	17–18		Experienced rowers (competing in Youth Olympic Games)
Filipas et al. [67]	Rowing	1500-m	18 (11 male)	11 ± 1.06		Experienced rowers (1.5 ± 0.85 years of rowing experience)
Kennedy and Bell [68]	Rowing	2-km	38 (19 male)		Two trials, a 10-week training program (4 rowing, 2 strength sessions per week) apart	A mixed group of experienced (> 1 year) and novice (< 1 year) rowers
Dimakopoulou et al. [69]	Rowing	2-km	15 (15 male)	15.37 ± 1.34		Experienced rowers (training seven sessions per week)
Schabort et al. [70]^a^	Rowing	2-km	8 (8 male)	16.0 ± 0.7	Three trials, 3 days apart	Novices
Hanon and Gajer [71]	Running	400 m	30 (15 female)			∙ World-class ∙ National ∙ Regional
Blasco-Lafarga et al. [33]	Running	600-m and 2 × 4 × 200-m	9 (9 male) 10 (10 male)	17.00 ± 0.66 25.29 ± 4.32		∙ More experienced ∙ Less experienced
Micklewright et al. [72]	Running	450-m 600-m 750-m 900-m	26 (15 male) 29 (15 male) 27 (14 male) 24 (16 male)	5.6 ± 0.5 8.7 ± 0.5 11.8 ± 0.4 14.0 ± 0.0		Novice schoolchildren
Chinnasamy et al. [73]	Running	750-m	36 (19 male)	12.6 ± 0.5	Two trials	Novice schoolchildren
Lambrick et al. [74]	Running	800-m	13 (8 male)	10.3 ± 0.7 (male) 10.6 ± 0.5 (female)	Four trials, on four separate days	Novice schoolchildren
Green et al. [75]	Running	3 × 800-m	12 (?) 16 (?)			∙ Collegiate ∙ Recreational
Watkins et al. [76]	Running	4-min (~ 1100 m)	10 (5 male)		Five trials, at least 3 days apart	Experienced (recreational level, training 4 ± 1 run sessions/week)
Diaz et al. [77]	Running	3-km	9 (9 male) 6 (6 male)	15.2 ± 0.7 24 ± 5.6	Two trials, one competitive season apart	∙ More experienced (8.1 ± 2 years) ∙ Less experienced (18 ± 8 months)
Deaner and Lowen [78]	Cross-country running	5-km	3948 (2032 male)	14–19		Experienced (competing in Virginia State Championships 5 km meet)
Stevens et al. [79]	Running	5-km	17 (17 male)		Two trials, 5–10 days apart	Experienced
Couto et al. [80]	Running	10-km	19 (19 male)	15 ± 2		Experienced (36 months (12–48 months))
Knechtle et al. [81]	Running	42.2-km	1 (1 male)	15.3		Experienced
Santos-Lozano et al. [82]	Running	42.2-km	190,228 (129,912 male)			∙ More experienced (professionals) ∙ Less experienced (amateurs)
Trubee et al. [83]	Running	42.2-km	32,121 (20,053 male)			∙ Experienced (elite) ∙ Less experienced (non-elite)
Deaner et al. [84]	Running	42.2-km	2929 (1676 male)			∙ More experienced runners ∙ Less experienced runners (total number of races, total number of marathons, personal bests for the 5 K and marathon, and earliest year with a recorded race)
Morais et al. [85]	Swimming	50-m	86 (86 male)	15–18		Experienced (European Junior Championships)
Dormehl and Osborough [86]	Swimming	100-m and 200-m	112 (56 male)	14.44 ± 0.69 16.98 ± 0.84		Experienced (competing at international schools swimming championships, 49.6- 96.8% of the junior world record)
Skorski et al. [87]	Swimming	200-m and 400-m and 800-m	16 (9 male)	16.9 ± 2.1	Two trials, 7 days apart	Regional to national level (training 34.7 ± 5.6 km/week)
Skorski et al. [88]	Swimming	400-m	15 (10 male)	18 ± 2 (14–23)		Competing at national level or higher (≥ 4 years of training)
Turner et al. [89]	Swimming	7 × 200-m incremental test	8 (8 male)	15 ± 1	Four trials, 1 week, 9 weeks and 20 weeks apart	Competing at national level (> 6 years of training)
Scruton et al. [90]^a^	Swimming	7 × 200-m incremental test	15 (?)	15 ± 1.5	Two trials, within 3–4 days	Regional level (26–33 km/week)
McGibbon et al. [91]	Swimming	1500 m	89 (89 male) 102 (102 male) 70 (70 male) 67 (67 male)	< 17 18–19 20–21 22 <		Experienced (top 100 of FINA world rankings)
Barbosa et al. [92]	Triathlon	Sprint Olympic	5902 (3196 male) 3314 (2225 male)	17 ± 2 21 ± 2		Experienced (World Triathlon Series)
Wiersma et al. [93]	Long-track speed skating	1500-m	104 (104 male)	15.25 ± 0.55 17.25 ± 0.55 19.25 ± 0.55	Five competitive seasons (three measurement points)	Experienced (8–20 races at the start of the study) ∙ More experienced ∙ Less experienced (1500-m races completed)
Stoter et al. [94]	Long-track speed skating	1500-m	120 (56 male)	17.6 ± 1.1	To trials, at least one year apart (subgroup of 12 [7 male] skaters were included in longitudinal analyses)	Experienced (national and international level)
Menting et al. [95]	Short-track speed skating	1500-m	224 (72 male) 1256 (665 male) 1687 (1132 male) 6556 (2691 male)	< 17 < 19 < 21 Senior (> 21)		Experienced (competing in junior and senior international competitions)
Menting et al. [96]	Short-track speed skating	1500-m	140 (140 male)	15.19 ± 0.26 16.11 ± 0.29 17.05 ± 0.29 18.03 ± 0.31 18.97 ± 0.31 19.56 ± 0.03	Six competitive seasons (six measurement points)	Experienced (competing in the Junior World Championships)
Sollie et al. [97]	Cross-country skiing	4.3-km 13.1-km	11 (11 male) 8 (8 male)	14.4 ± 0.5 22.6 ± 4.3		Experienced (national level)
Formenti et al. [98]	Cross-country skiing	10-km	11 (11 male)	16.45 ± 1.67		Regional and national (training 12–15 h/week)
Carlsson et al. [99]	Cross-country skiing	90-km	9691 (8788 male)			∙ More experienced (> 3 years) ∙ Less experienced (< 4 years)
Alves et al. [100]	Race walking	10-km and 20-km	29 (14 males)			∙ More experienced (49–240 months) ∙ Less experienced (5–48 months)
Sealey et al. [101]	Outrigger canoeing	1-km	11 (0 male)		Four trials within 2 weeks	Experienced rowers (> 2 years) (training 4–11 sessions/week)
Moss et al. [102]	Cross-country Mountain biking	86-km	8182 (7178 male)	16.5–65 +		∙ More experienced ∙ Less experienced cyclists (previous races completed)
Sampson et al. [103]	Rugby league	24-min small sided games	16 (16 male)	14.9 ± 0.5		Amateur level
Johnston et al. [104]	Rugby league	Tournament	28 (28 male)	16.6 ± 0.5		Amateur level
Christi et al. [105]	Cricket	14 shuttle sprints	24 (24 male)			∙ More experienced (early in batting line-up) ∙ Less experienced (late in batting line-up)
Moss and Twist [106]	Handball	Repeated Shuttle-Sprint and Jump Ability test	8 (8 male)	16.1 ± 1.0		National level
Burdon et al. [107]	Exercise circuit	Tasks 1–4: < 15-min Tasks 5–6: < 2 min	35 (17 male)		Three trials, within 10 days with 24 h separating each trial	Novice
Gross et al. [108]	Alpine skiing	90-s box jump test	9 (9 male)	16.8 ± 1.3 (range 16–18)	Two trials, a 8-day HIT block comprising of 10 sessions, apart	Experienced (from an elite sports school)
Reid et al. [109]^a^	Elbow flexion maximal voluntary contractions	12 × 5 s	14 (0 male)	15.2 ± 2.1		Novice

a = included additional to the literature search. ∙ = comparison between groups of different experience levels. * = Age only reported for studies investigating individuals younger than 18 years of age

## Results

The articles included in this evaluation comprise a broad selection of exercise tasks and research designs (Table 1). The majority of the articles investigated tasks related to endurance sports, encompassing cycling (*n* = 19, distance: 1500 m–20 km), running (*n* = 16, distance: 400 m–42.2 km), rowing (*n* = 6, distance: 1–2 km), swimming (*n* = 7, distance: 50–1500 m), speed skating (*n* = 4, distance: 1500 m) cross-country skiing (*n* = 3, distance: 4.3–90 km) or another endurance sport (*n* = 4). Team sports were investigated in two articles. The other studies investigated individuals performing an exercise circuit (*n* = 2), a repeated jumping (*n* = 1), sprinting (*n* = 1) or resistance (*n* = 1) task. In total, 41 studies investigated pacing behaviour in a controlled laboratory or field setting, whereas 21 articles investigated the pacing behaviour of athletes during competition. Two studies combined both study designs.

### The Development of Pacing Behaviour in Individuals Under 18 Years of Age

A total of 33 included articles reported on the pacing behaviour of individuals under the age of 18 years, distributed between the age ranges of 5–10 (*n* = 1), 10–14 (*n* = 4) and 14–18 (*n* = 28) years old (Table 1). Six studies compared the pacing behaviour of children and adolescents of differing ages, four of which used a cross-sectional design [72, 86, 91, 95] and two used a longitudinal design [93, 96].

When cross-sectionally comparing the pacing behaviour of schoolchildren, performing a 3–4 min running task, the groups with an age averaging 5.6 and 8.7 years old exhibited an all-out pacing behaviour, characterized by a fast start and a gradual decline in speed until the end of the task [72]. Conversely, groups of older children (averaging 11.8 and 14.0 years old) exhibited a parabolic distribution of effort, with a fast start and an end-spurt finish but a relatively slower middle section. This parabolic distribution has also been reported by two other articles studying the pacing behaviour of children between 10 and 13 years old, performing similar exercise tasks [73, 74]. Four included studies compared the pacing behaviour of adolescents and adults, performing middle-distance tasks of running [33, 77], swimming [91], and cross-country skiing [97], reporting that adolescents exhibited a parabolic distribution of effort, whereas adults exhibited a more even pace. Indeed, when long-track speed skaters competing in a 1500-m race were longitudinally measured at 15, 17 and 19 years of age, the skaters exhibited a development of pacing behaviour towards that of adult skaters, characterized by a relatively slower start and faster middle section [93].

Parallel to development in the distribution of effort itself, the influence of environmental factors on pacing behaviour seems to develop with age. The presence of other exercisers was reported to have a detrimental effect on exercise performance in younger children (10.3 ± 0.7 years old) [74], no effect in adolescents (15.8 ± 1.0 years old) [49] and resulted in an improvement in performance in adults [50]. An alteration of pacing behaviour was reported to be the cause of the change in exercise performance [50, 74]. Further corroboration was provided by two studies investigating short-track speed skating, a head-to-head type of competition featuring a highly interactive environment [95, 96]. Throughout adolescence, short-track speed skaters seemed to develop both their positioning during the race as well as their capability to preserve energy during the initial phase of the race [95, 96]. These adaptations indicate an improved integration of environmental factors in the athletes’ pacing behaviour and are linked to a higher velocity during the critical final laps resulting in improved race performance [110].

### The Effect of Experience on Pacing Behaviour

The effect of prior experience on pacing behaviour has been investigated in thirteen included articles (Table 1) by means of comparing the pacing behaviour of adult exercisers of differing levels of experience performing a predetermined exercise task. More experienced exercisers are not only better able to exercise at a prescribed pace [75], but also exhibit a pacing behaviour more suited to the needs of the specific exercise task. Generally, this is expressed as an all-out behaviour during short tasks [71] (< 120 s), or a more even pacing behaviour during longer exercise tasks [82–84] (> 120 s). Experienced individuals are also able to successfully incorporate environmental factors (e.g. terrain) into their pacing behaviour, aiding their performance [99, 102]. Novice exercisers seem to prefer information regarding task completion (distance) and mainly report dissociative, outward monitoring thoughts [57, 58]. Experienced exercisers, on the other hand, prefer information concerning task performance (speed) and primarily report associative, task-focused thoughts (concerning power and cadence) [57, 58].

A total of seven articles reported on the acquisition of pacing behaviour through repeatedly exposing adult novices to the same exercise task. Two of these studies incorporated a training program between repeated bouts of exercise [65, 68]. All seven studies involved exercise tasks with a duration longer than 120 s (minimum: 189.4 s [48], maximum: 2708.35 s [62]), and all reported a change in pacing behaviour with repeated task exposure. Within three studies this was expressed by a decrease in effort during the first section of the task and an increase in the final section [48, 52, 62]. Four studies reported an increase in effort during the initial and middle sections and a decrease during the final section [57, 65, 68, 107]. Two studies reported that the adaptation of power output distribution during consecutive tasks was paralleled by the anaerobic energy expenditure and blood lactate levels, indicating a change in the management of energy reserves over the bout duration [48, 65]. Lastly, the manipulation of the effect of gaining experience on pacing behaviour by interventions such as withholding information on task duration [53], providing only a half familiarization [62], or withholding duration feedback or providing false feedback during the trial (5% improvement compared to actual performance) [61], lead to a maladaptation of pacing behaviour and a decrease in exercise performance.

## Discussion

This review provides the first consolidated evidence that pacing behaviour in exercisers up to 18 years old develops with age. This demonstrates that pacing behaviour development starts in childhood and continues through adolescence. All included studies examining the effect of repeated task exposure on pacing behaviour in adults (*n* = 7) support the hypothesis that pacing behaviour is acquired through the gathering of exercise task experience, similarly to other skilled behaviour. Manipulation of the skill acquisition process by interfering with the gathering of task experience results in a maladaptation of pacing behaviour (*n* = 3). It is therefore apparent that pacing is similar to other skills, in so far that it has a developmental pathway and appears not to reach maturity until adulthood, by which time there is still capacity to further improve through task experience.

### Pacing Behaviour Development

The characteristics of pacing behaviour among young children (< 9 years old) tend to manifest in inconsistent approaches to the task demands, encompassing both the task characteristics (e.g. workload) and the environment (e.g. other competitors). An example is the adoption of an all-out approach of maximal effort until fatigue in an exercise task lasting over 120 s, in which an even distribution is known to lead to better performance [111, 112]. However, with age, a development towards a parabolic distribution of effort is evident in tasks with similar demands. This parabolic pacing behaviour includes a conservation of effort at the start of the exercise, reflecting an increased involvement of goal-directed decision-making regarding effort distribution. The development of pacing behaviour continues during adolescence, with the manifestation of pacing behaviour which increasingly fits the task demands. As part of this development, exercisers are not only able to pace their efforts during an isolated time trial event, but also in complex situations in which environmental factors (e.g. opponents) need to be taken into consideration [95, 96].

Given that pacing behaviour is similar to other skilled behaviour, it is likely that the origin of the development of pacing behaviour can be traced to primary features of childhood and adolescence: physical maturation and cognitive development. Indeed, various physical maturity milestones, such as the growth spurt and the trajectory of muscle mass development [26–29], seem to match the roadmap of pacing behaviour development, as described above. Unfortunately, only four included studies reported on the pacing behaviour of children within the age-range of the growth spurt (10–14 years old) and none of these articles reported on the relationship between a measure for physical maturation and pacing behaviour [72–74, 86]. With regard to cognitive development, Micklewright et al. [72] reported the same development of pacing behaviour could be found based on children’s ages as based on children’s scores for cognition, as measured by Piaget’s stages of intellectual development. It could therefore be proposed that pacing behaviour development is linked not specifically to age, but rather to the rate of cognitive development. Comparing the stages of cognitive development proposed by Piaget to the roadmap of pacing behaviour development during childhood and adolescence strengthens this hypothesis. Piaget’s third stage (i.e. concrete operational stage) spans the ages 7–11. During this stage, children gradually gain the capability to concentrate on more than one aspect of a problem simultaneously and mentally represent actions or events based on previous experience [24]. These mental capabilities could provide children with the aptitude to recall and appreciate that making decisions regarding effort distribution before and during exercise (i.e. a conservation of effort during the opening phase of the exercise), could improve their overall task performance. However, children at this stage are limited to pondering situations that are real or based on their own experiences [24]. This could explain why the presence of opponents has a detrimental effect on the pacing behaviour of children [74], as the presence of opponents likely provides a stronger stimulant than the abstract notions of hypothetical future performance improvement, afforded by adopting a slower pace. Piaget’s fourth stage of cognitive development (i.e. formal operational stage) ranges from 11 to 20 years old [24]. During this stage, individuals gain the capability of considering ideas that are not based on reality, observable objects or experience-based thoughts [24]. Additionally, individuals acquire the aptitude to systematically generate and consider multiple possible solutions to a problem [24]. These mental capabilities provide a better grasp on the hypothetical future rewards from pacing one’s efforts and likely underpin the continuation of the development of pacing behaviour throughout adolescence. Furthermore, these cognitive capabilities facilitate the adaptable pacing behaviour needed in complex competitive environments and therefore enable the integration of environmental factors (e.g. opponents) into the development of pacing behaviour, which occurs during adolescence.

### Acquisition of Skill Pacing

**Fig. 2:**
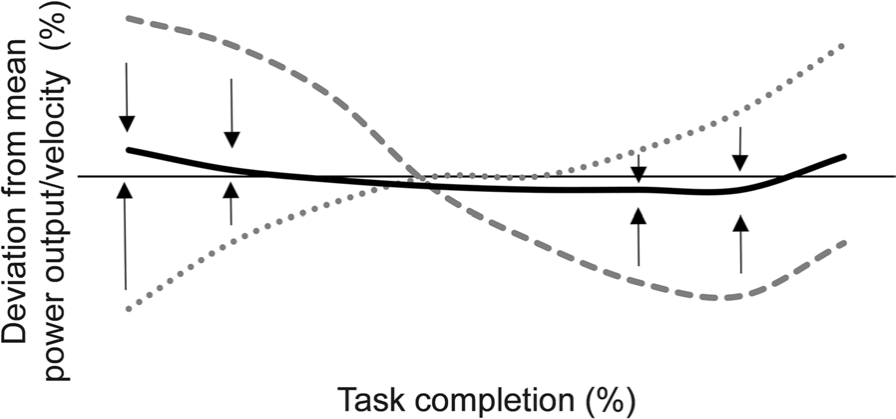
Example of repeated exercise task exposure affecting the pacing behaviour of novice adult exercisers. Grey dotted: exercisers initially exerting not enough effort, grey striped: exercisers initially exerting too much effort, black bold solid: more even pacing behaviour. Arrows: change with increased exercise task experience. The horizontal solid line represents the mean power output/velocity. This example is based on the collective results of included studies that reported on the change in pacing behaviour resulting from repeatedly exposing adult novices to the same exercise task (> 120 s)

The current study is the first to investigate the available literature for the effect of experience on pacing behaviour. From the consolidated literature, it can be concluded that there is an evident effect of gathering experience on pacing behaviour across exercise types and durations. More experienced exercisers are not only better at adopting a prescribed pace but also exhibit a pacing behaviour that better suits the task demands and competitive environment. All included studies featuring repeated tasks revealed that with experience, novice exercisers adapt their pacing behaviour. Although the direction of change seemed to ostensibly differ between studies, collectively all studies reported a change towards a more even distribution of effort as experience increased (Fig. 2). Within skill learning literature the behaviour of novices is characterized by large errors and relatively large corrections for these errors [25]. As the learning process proceeds, individuals will learn to match the task stimuli and their actions with a resulting task performance [19, 25]. Similarly, the proposed explanation for the effect of experience on pacing behaviour is a reduction of the mismatch between the exerciser’s individualized performance capabilities and the task demands, encompassing both the task characteristics (e.g. workload) and the environment (e.g. terrain) [48, 53, 61, 62, 65, 105, 107]. This mismatch results in the exerciser exerting too much effort (i.e. overestimation of the exerciser’s performance capabilities or underestimation of the task demands) or not enough (i.e. underestimation of the exerciser’s performance capabilities or overestimation of the task demands). As the pacing process is continuous, repeating mismatches between stimuli and action can result in an undulating pace over the course of a task. Unnecessary accelerations and decelerations are detrimental to performance, as even minor fluctuations in velocity result in a greater overall energy cost [111]. However, as the task is repeated, exercisers learn to associate the stimuli (e.g. task demands and afferent signals) and actions (i.e. continuing, increasing or decreasing the exerted effort) with the resulting task performance. This knowledge results in more informed decision-making, reducing the occurrence of inefficient adoptions of overly aggressive or conservative pace. Within tasks longer than 120 s, this results in a more even distribution of effort, which is linked to increased task performance [111, 112].

Furthermore, within skill acquisition literature, it is stated that as individuals gain more experience with a task, less attention is needed for the same level of task performance, allowing for secondary tasks to be performed simultaneously [19, 113]. The possession of residual attention capacity could explain why more experienced exercisers are able to process and integrate environmental factors, such as the terrain and the behaviour of opponents. Additionally, the skill acquisition literature states that the main goal for novices is to achieve a crude level of success in a task [19, 40]. Indeed, as novices lack a reference point for the workload required for a specific exercise task, novice exercisers are thought to have the primary goal of finishing the exercise without lasting negative consequences [57, 65]. To reach this goal, novice exercisers mainly acquire information regarding task completion (e.g. elapsed distance or time) [57] and concentrate on dissociating themselves from the afferent signals of fatigue (e.g. pain and discomfort) by means of outward thought [58]. Although this might help novice exercisers complete the exercise task, it also hinders their capability to match their afferent signals to the task demands [114]. Alternatively, experienced exercises have the knowledge that they are able to finish the task (without lasting negative consequences), which allows them to set the goal of realizing the best possible task performance. Experienced exercisers therefore direct their thoughts towards, and gather information about, factors relating to their task performance (e.g. power, cadence and speed) [57, 58].

A point should be made, that as each individual’s performance capabilities differ and the task demands remain constant, optimal pacing behaviour will slightly differ between individuals [115]. This variation between individuals is likely the cause of the variation of pacing behaviour between athletes at the elite level [116, 117]. The acquisition process, as described above, results in a better match of an individual's performance capabilities to the task demands, facilitating a more appropriate pacing behaviour and improving task performance. It is through repeating and optimizing this acquisition processes that the match between individual performance capacities and task demand is perfected, and an individual's pacing behaviour is optimized.

### Practical Applications

It is assumed that pacing is evident in almost all non-reflex physical activity and that it is fundamental to the successful completion of physical tasks [1]. Given this, adequate pacing behaviour development could provide a feeling of competency, allowing for more task enjoyment and inhibition of drop-out from physical activity, with all the associated health benefits [1, 118]. Although in the current study, skill development and acquisition have been treated as separate processes, it is evident that gathering experience is a key factor in skill development [24]. From this stems the notion that children and adolescents should be provided with the opportunity to practice exercise tasks to optimally facilitate skill development [24]. Four out of five studies included in this review that investigated children and adolescents during repeated tasks, reported a change in pacing behaviour and/or an improvement exercise task performance [49, 70, 74, 87]. When asked to estimate the completion time of a 2-km cycling time trial before starting the trial, novice adolescent exercisers reported a significant (*p* < 0.05) difference between the expected (453.00 ± 249.18 s) and actual (240.50 ± 27.37 s) finish time during the first trial [49]. However, the gap between the expected finish time and the actual finishing time decreased by 66.2% after the first trial, indicating a better matching of performance capabilities and task demands, as task experience increased. These findings emphasize the importance of providing children and adolescents with the opportunity to gather exercise experience in order to develop their pacing behaviour over time. However, it is important to acknowledge that even with ample practice, variation in physical maturity and cognitive development will constitute some children to be able to adequately distribute their efforts at a relatively young age, whereas others might not exhibit this behaviour until they are much older. Coaches and parents are therefore advised to monitor the level of pacing behaviour development and gradually incorporate increasingly challenging pacing exercises during the course of childhood and adolescence, in order to support the development of pacing behaviour.

To facilitate and optimize the pacing skill acquisition, lessons from the skill acquisition literature suggest that exercisers should start with establishing a stable behaviour [119, 120]. It is suggested that in novice adults a relatively stable pacing behaviour occurs after three or four sessions of repeated task exposure [62, 65, 121]. However, this number could increase as variation in task demands increases (e.g. a highly interactive competition environment). After establishing a stable pacing behaviour, intervention-induced variability could provide exercisers with opportunities to test variants of their established pacing behaviour, enlarging their familiarity with the association between incoming stimulants, decisions made and the resulting task performance [25, 122]. This could lead to the discovery of a more fitting pacing behaviour for the specific exercise task. In addition, variable practice could lead to a greater generalization and flexibility of the exercisers’ pacing behaviour, allowing them to respond better to novel situations (e.g. the behaviour of competitors) [119]. Lastly, the provision of augmented feedback could also be used to adapt the difficulty of the task in order to provide an adequate challenge and optimize learning [120]. When practising the same task, novice exercisers might be helped by providing frequent and immediate feedback, whereas experienced exercisers might be challenged by the decrease, delay, or removal of feedback.

### Future Directions

Although the match between milestones in pacing behaviour development and the changes in physical maturation and cognitive development form a logical framework, more (longitudinal) studies are needed to deepen the knowledge of the link between age, physical maturation, cognitive development and pacing behaviour development. Considering the relevant links between cognitive development and pacing behaviour development, a next step in research could be to dive deeper into which specific sections of cognitive functioning would underlie the development of pacing behaviour. Elferink-Gemser and Hettinga [18] previously proposed a model in which the pacing process mirrors the self-regulatory process, and suggested that improvement in meta-cognitive functions could be underpinning the development of pacing behaviour within childhood and adolescence [18]. Indeed, meta-cognition has been shown to be under development during childhood and adolescence [123, 124], positively related to exercise performance [125, 126] and can be measured by validated instruments [124, 127]. Future experimental research could therefore be done to find whether the development of meta-cognitive functioning indeed is part of the underlying mechanism of pacing behaviour development. It is evident that experience plays a key part in pacing behaviour development. Unfortunately, this relationship is often oversimplified in the literature, as researchers assume a strictly causal relationship between age and experience. By uncoupling experience and age, future research could further unravel the intricate role of experience within pacing behaviour development. Furthermore, it has been proposed previously that acquiring the skill to pace an exercise task is facilitated by the acquisition of other skills, including accurately perceiving time [128], inhibiting distracting stimuli [59], as well as planning and evaluating [18, 129]. Investigating the relationship between these other skills and pacing could provide a better understanding of what it takes to acquire this complex psychophysiological skill. Lastly, in the current review, the acquisition of pacing is most notably analysed by observing the effect of providing or manipulating experience on pacing behaviour. However, within the literature, definitions of skill acquisition commonly include the notion that learning has a relatively permanent effect on behaviour [19, 25]. To test this, experimental designs to test learning include retention tests. Within the current review, no studies were found that measured the retention of the skill after a period without practice. To further explore the effect of experience on pacing behaviour, future research designs should consider including retention tests.

## Conclusion

The current review aimed to investigate the development of pacing behaviour during childhood and adolescence as well as the acquisition of the skill through experience. This was achieved by assembling and analysing the (sport) scientific literature discussing the effect of age (up to 18 years old) and gathered experience on pacing behaviour. The findings of this study demonstrated the first consolidated evidence that children display an initial development of decision-making regarding effort distribution from around 10 years old, a development that continues in adolescence. Based on shared milestones, a case can be made that pacing behaviour development is underpinned by an interconnected relation of physical maturation, cognitive development and gathered experience. The skill to adequately pace exercise tasks could provide children with an increased sense of competence and enjoyment in physical activity and exercise, emphasizing the importance of monitoring and practising pacing exercise tasks during childhood and adolescence. Task repetition results in an adaptation of pacing behaviour towards the task demands, including task characteristics (e.g. workload) and the environment (e.g. terrain). These changes can be explained by knowledge from the skill acquisition literature: pacing behaviour is acquired because with experience (1) the match between stimuli, actions and task results improves, (2) attentional capacity is freed for secondary tasks, (3) the task goal switches from task completion to improved task performance. The resemblance between the development and acquisition of pacing to the same processes in other skills invites the practical application of established concepts in skill acquisition and development literature (e.g. intervention-induced variability and augmented feedback) to the field of pacing research. This integration provides the field with exciting future research questions as well as practical applications in physical education, healthcare, and sports.

## Data Availability

All data generated or analysed during this study are included in this published article.
